# Multi-Granular Embedding and Hybrid Encoder–Decoder Architecture with Temporal Linear Regression Bypass for Sensor-Based Time Series Forecasting in Smart Infrastructure

**DOI:** 10.3390/s26185953

**Published:** 2026-09-20

**Authors:** Hsu-Yung Cheng, Huan-Hsuan Lin, Chi-Lun Jiang, Chih-Chang Yu

**Affiliations:** 1 Department of Computer Science and Information Engineering, National Central University, Taoyuan City 320, Taiwan; 2 Department of Information and Computer Engineering, Chung Yuan Christian University, Taoyuan City 320, Taiwan; ccyu@cycu.edu.tw

**Keywords:** deep learning, time series forecasting, multivariate time series, periodic time series

## Abstract

In this work, we propose a robust deep encoder–decoder neural network that integrates multiple deep sequence models for time series forecasting in sensor-based applications. The proposed approach features a sophisticated input embedding layer that integrates scalar features, position encodings, and multi-granularity time embeddings. To improve computational efficiency, we employ a generative-style decoder inspired by the Informer architecture, which enables full-length sequence prediction in a single iteration, avoiding the latency associated with traditional autoregressive models. The architecture is highly flexible, supporting either Gated Recurrent Unit (GRU) or Transformer blocks as foundational layers to process latent temporal dependencies. Recognizing that purely nonlinear deep learning models often struggle to capture sharp, short-term local variations due to inherent input scale insensitivity, we augment our model with a parallel temporal linear regression unit. This bypass mechanism captures linear local trends, effectively combining the predictive power of nonlinear deep feature extraction with the sensitivity of linear regression. Experimental evaluations on the Electricity Consumption (EC) and Speed Index California (SIC) datasets demonstrate that the GRU-based implementation reduces the average Mean Squared Error (MSE) by approximately 22% and 16%, respectively, compared with the best-performing baseline across different output lengths. Ablation studies further confirm that the temporal linear regression bypass substantially improves forecasting accuracy, particularly on the EC dataset, where it provides an average MSE reduction of approximately 55%.

## 1. Introduction

Periodic time series are characterized by repeating patterns that cause samples to exhibit rhythmic fluctuations. The period of such cyclic patterns can manifest over any fixed time interval. Analyzing these series is often challenging, as they may be composed of multiple intertwined and superimposed patterns with distinct periodicities. This inherent complexity complicates the analytical process. However, by decomposing these underlying patterns and leveraging them effectively, prediction accuracy can be significantly improved. To achieve this, the model must have the ability to capture temporal dependence at different time scales so that it can gain insights into the hidden temporal structure of the time series.

In practical applications, the effectiveness of modern smart infrastructure, ranging from Smart Grids to Intelligent Transportation Systems (ITS), relies heavily on the continuous ingestion of high-fidelity data streams from distributed sensor networks. In the case of energy management, Advanced Metering Infrastructure (AMI) and smart meters function as the primary sensing nodes, providing high-resolution measurements of electrical load to facilitate grid stability and demand-response optimization. Similarly, traffic management systems utilize a combination of inductive loop detectors and mobile probe vehicle telemetry to sense real-time highway congestion and flow dynamics. These real-time series exhibit periodic characteristics. To exploit these underlying features, we often generate period-based predictions to facilitate effective long-term planning and management. Despite the richness of this sensor-derived data, forecasting algorithms frequently encounter challenges due to the stochastic nature of physical signals, including signal noise, sensor drift, and intermittent connectivity.

Consequently, our objective is to explore robust methods for accurately forecasting long-term periodic time series and to evaluate their performance in practical real-world scenarios. However, standard deep learning backbones face inherent architectural limitations when addressing these robust forecasting needs. While complex deep learning models excel at capturing intricate nonlinear relationships, standard recurrent and Transformer architectures frequently struggle with fitting global linear trends and static magnitude baselines. When processing non-stationary time series, neural backbones are prone to nonlinear drift and over-smoothing near extrema. To address this limitation, we introduce a Linear Representation (LR) bypass branch within our framework. Driven by this explicit trend–residual decoupling motivation, we propose a hybrid architecture that integrates a lightweight recurrent encoder with a non-autoregressive attention-based decoder, augmented by a multi-granular temporal embedding module and a parallel linear regression bypass. In addition, the input-scale insensitivity of nonlinear deep learning models provides another important motivation for the proposed linear bypass. The output range of nonlinear deep learning models may be less responsive to changes in the input scale, which can reduce forecasting accuracy when the time series contains drastic short-term variations. Therefore, improving the model responsiveness to local magnitude changes is important for accurately capturing short-term fluctuations while preserving long-term temporal patterns. This configuration not only extracts complex nonlinear latent patterns but also preserves the sensitivity to short-term signal fluctuations inherent in high-frequency sensor readings, thus providing a more resilient forecasting framework for real-world sensing applications.

## 2. Related Works

Statistical methods such as Autoregressive Integrated Moving Average (ARIMA) [1] and Exponential Smoothing (ES) [2,3] are two foundational frameworks in traditional time series forecasting. ARIMA is an extension of Autoregressive Moving Average (ARMA) and employs differencing to make the input sequence stationary. ES is a heuristic forecasting model that computes the weighted average of past observations, where the weights decay exponentially as the time interval from the prediction increases. Traditional models often integrate time series decomposition techniques [4,5] to preprocess input sequences, decomposing them into multiple components based on their distinct characteristics. These components are then modeled individually to enhance overall accuracy and robustness. The advantages of traditional statistical methods lie in their simplicity. However, the linear assumptions often limit their ability to capture complex nonlinear features.

In contrast, machine learning methods offer enhanced modeling flexibility compared to traditional statistical approaches. They often possess the capacity to capture complex nonlinear features and dependencies. Methods such as decision trees [6], Support Vector Regression (SVR) [7,8], and Random Forest Regression (RFR) [9] have been shown to outperform traditional statistical models in various applications. However, these methods often necessitate domain expertise for feature engineering, which limits their generalizability across diverse datasets. Furthermore, the inherent structural limitations of these machine learning methods make long-term time series prediction still challenging.

With the vigorous research on deep learning, many studies show that deep learning-based approaches can achieve astonishing results in many fields. In the field of long-term time series prediction, Recurrent Neural Network (RNN)-based models [10,11] are often used to extract long-term temporal dependencies and nonlinear features of various time series, and have achieved good results in multiple tasks. DeepAR [12] generates predictions in an autoregressive manner and models the probability distribution of the predicted values using Long Short-Term Memory (LSTM) [13] to provide a measure of uncertainty for the predictions.

The main characteristic of Convolutional Neural Network (CNN) architecture in deep neural network models when processing time series data is its ability to effectively extract regional features. Although CNNs are mainly used for local feature extraction in time series, with proper parameter or architecture adjustments, CNNs can also learn very long-term temporal dependencies.

Hybrid models are those combining different networks to enhance their ability to acquire different-scale features. These models consist of multiple components, and each component is responsible for processing different features. Merging them together helps models obtain a more comprehensive feature representation. LSTNet [14] uses a CNN to capture preliminary local features of the input series, and uses LSTM with different time intervals to capture long- and short-term dependencies. MLCNN [15] is an encoder–decoder model taking the concept of Construal Level Theory (CLT) by stacking multiple convolutional layers to obtain regional features at different abstraction levels.

Attention-based architectures, most notably the Transformer [16], have gained widespread adoption in time series forecasting due to their constant path length for internal sequence connections and inherent parallelizability. To address the computational bottlenecks associated with standard self-attention, Informer [17] proposes ProbSparse self-attention using Kullback–Leibler (KL) divergence to measure the importance of every query to reduce spatial complexity and also introduces a generative-style decoder to improve prediction efficiency. Building upon these foundational designs, recent advanced frameworks have evolved rapidly to tackle complex temporal dependencies and multi-granularity representations through architectural decomposition, frequency domain analysis, and hierarchical attention. Specifically, Autoformer [18] replaced traditional self-attention with auto-correlation mechanisms paired with progressive series decomposition to isolate seasonal and trend components effectively. FEDformer [19] further enhanced long-range representation by executing attention in the frequency domain via Fourier and Wavelet transforms, despite incurring non-trivial mathematical overhead. To better capture multi-scale features, contemporaneous architectures such as the Granularity Fusion Transformer (GFT) [20] and Pyraformer [21] explicitly model fine-to-coarse temporal relationships through multi-granularity patching or hierarchical attention modules. Recently, advanced time series forecasting frameworks have evolved rapidly to address complex temporal dependencies and multi-granularity representations.

To mitigate the high computational cost of attention mechanisms, linear and multi-periodic paradigms emerged. DLinear and NLinear [22] demonstrated that simple decomposed linear projections could outperform complex Transformers, highlighting the risk of over-parameterization in long-sequence forecasting. TimesNet [23] expanded 1D time series into 2D spaces to capture intraperiod and interperiod variations simultaneously using 2D convolutions, albeit introducing multi-scale architectural complexity. Scaleformer [24] introduced an iterative multi-scale refining framework that progressively enhances predictions across temporal scales, though its multi-step refining pipeline increases inference latency. To further exploit fine-grained temporal structures and channel dependencies, patch-based and specialized multi-scale architectures were subsequently developed. PatchTST [25] segmented time series into sub-series patches and adopted channel-independence, significantly boosting prediction accuracy and reducing computational overhead. However, patch segmentation may unintentionally disrupt fine-grained cross-step continuity. Crossformer [26] introduced a two-stage attention mechanism to explicitly capture both cross-time and cross-dimension dependencies, albeit at the expense of a higher memory footprint when scaling to high-dimensional datasets. More recently, MSFformer [27] employed multi-scale feature extraction to model periodic pattern dynamics, yet its performance remains highly sensitive to input normalization schemes. Its unnormalized feature projections limit its competitive performance under standard benchmark evaluation. Similarly, FCP-Former [28] integrated frequency compensation modules with patch-based embeddings to mitigate information loss from temporal slicing, though the additional spectral transformations introduce non-negligible feature alignment overhead. Recent state-of-the-art literature has explored inverted dimensions, lightweight representations, and novel state space backbones. While these state-of-the-art methods achieve rich multi-scale or channel-wise representations, they often introduce substantial model complexity, heavy architectural overhead, or hyperparameter sensitivity. On the other hand, existing lightweight linear variants often struggle to capture nonlinear local fluctuations. In contrast, our approach prioritizes a lightweight additive embedding paired with a parallel linear bypass, achieving an optimal balance between low computational overhead and sharp local sensitivity.

To sum up, deep learning methods show great promise for addressing long-term time series prediction problems due to their high generalization and adaptability. This is particularly advantageous when dealing with input sequences that possess complex features and nonlinear temporal relationships. In this paper, we leverage the power of CNN for initial local feature extraction from the input sequence together with global date–time information presented by the proposed time embedding method to form the representation of the input sequence. This representation is then fed to our encoder–decoder model implemented by different sequence models, such as GRU or Transformer, to generate final predictions.

## 3. Proposed Multi-Granular Embedding and Hybrid Encoder–Decoder Architecture

**Figure 1:**
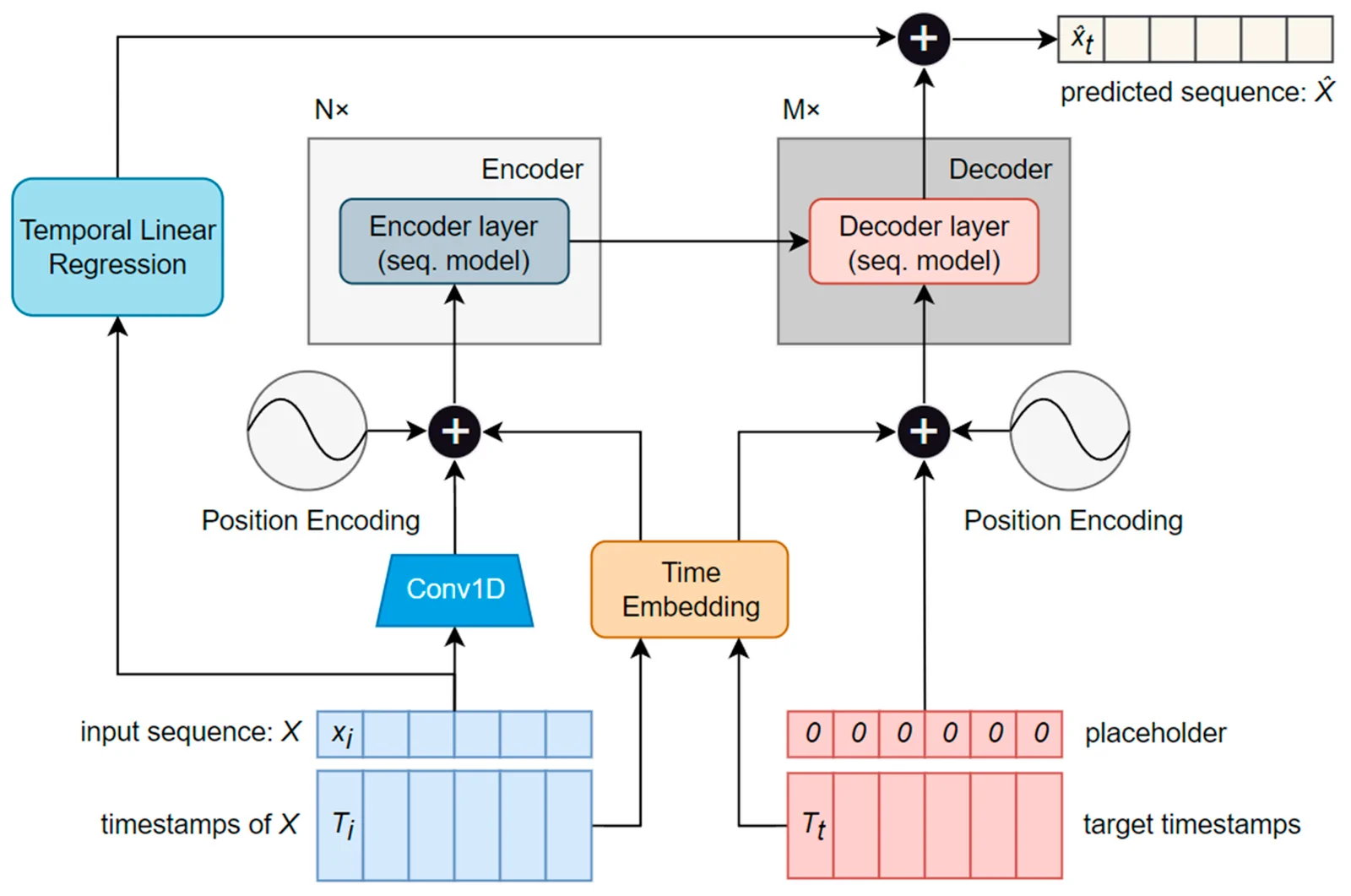
Overview of proposed framework.

In this section, we present the detailed design of our proposed forecasting framework. The hybrid nature arises from coupling a lightweight sequential recurrent encoder or self-attention encoder with a generative attention decoder. This neural backbone is further augmented by a multi-granularity temporal embedding (TE) module and a parallel linear representation (LR) bypass for explicit trend–residual decoupling. The overview of the proposed framework is illustrated in Figure 1. The problem formulation, input embeddings, network architecture, and temporal linear regression are elaborated in the following subsections.

### 3.1. Problem Formulation

**Figure 2:**
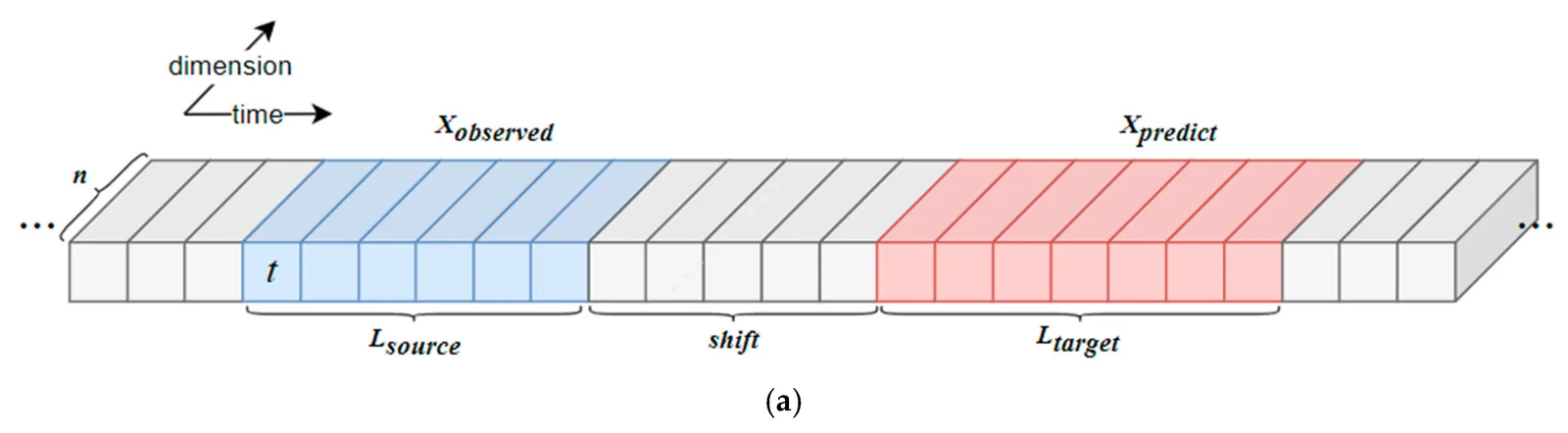

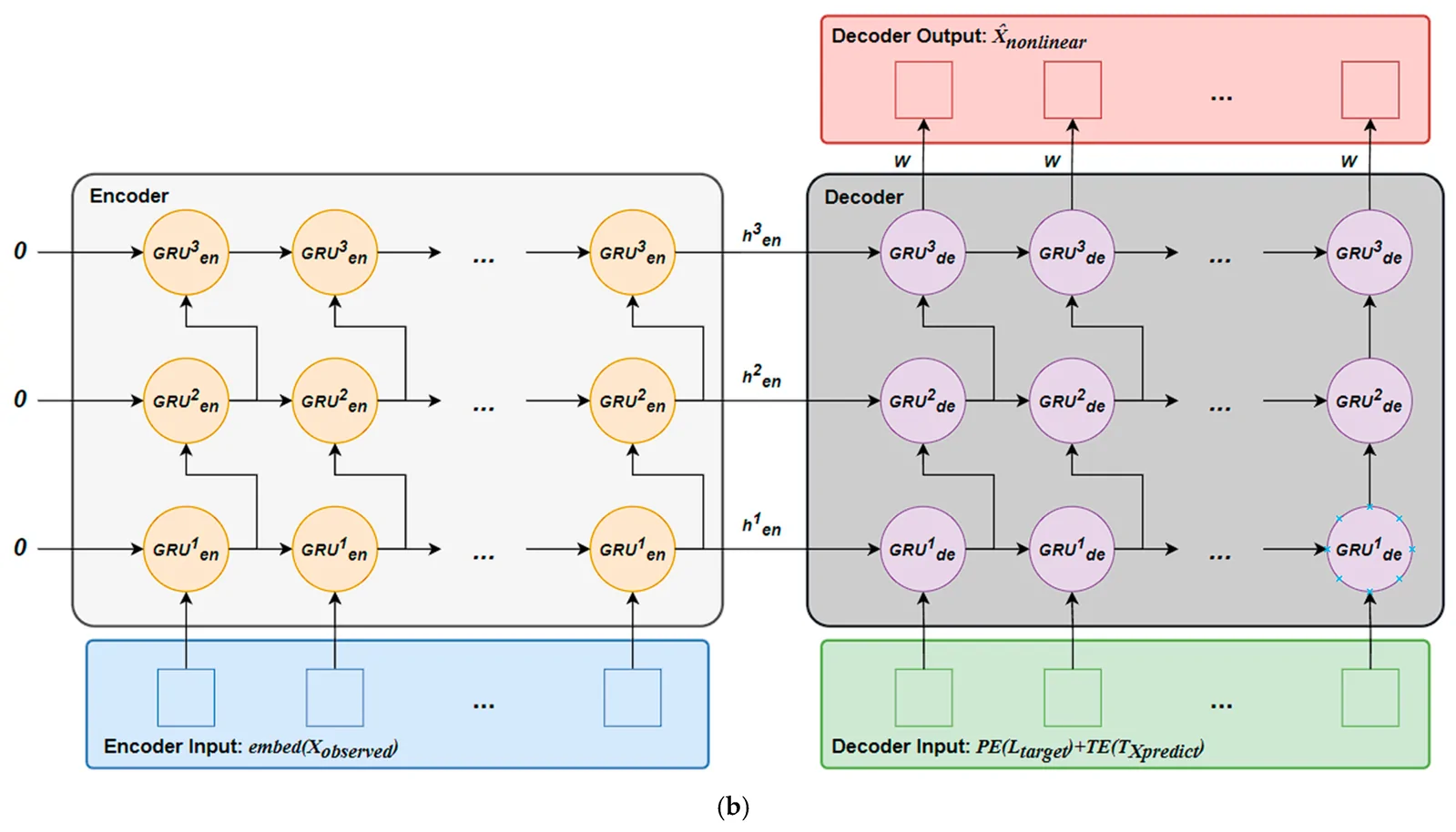
Visualization of (**a**) the definition of a series; (**b**) the GRU encoder–decoder model in the proposed system.

For any given real-time series $X=\left\{x_{i}\in R^{n}:\;i\in N\right\}$, the input series of length $L_{source}\in Z^{+}$ starting at time t∈N is denoted as $X_{observed}=\left\{x_{i}:\;i\in \left\{t,\ldots ,t+L_{source}-1\right\}\right\}$, while the target series of length $L_{target}\in Z^{+}$ is denoted as $X_{predict}=\left\{x_{i}:\;i\in \left\{t+L_{source}+s,\ldots ,t+L_{source}+s+L_{target}-1\right\}\right\}$. The temporal distance between $X_{observed}$ and $X_{predict}$ is called “shift” and denoted as s∈N. Figure 2a gives a visual explanation of the series’ definitions.

### 3.2. Input Embeddings

The input embeddings consist of three parts: scalar embeddings, position encodings and time embeddings. For a given input series, these three components are combined to form its representation as follows:

(1) $$Embed\left(X\right)=Conv1D\left(X\right)+PE\left(L_{X}\right)+TE\left(T_{X}\right)$$

where X denotes the input time series, $L_{X}$ denotes the sequence length of X, and $T_{X}$ represents its corresponding timestamps. Conv1D(·) denotes the scalar embedding obtained through a 1-D convolution, while $PE\left(\cdot \right)$ and $TE\left(\cdot \right)$ denote the positional encoding and temporal embedding, respectively.

Scalar embeddings is a 1-D convolutional layer with a stride of 1 and activation of Exponential Linear Unit (ELU), aiming at extracting local patterns of input series. We also introduce the position encoding method first proposed by Vaswani et al. [16] to present inter-position information. Lastly, inspired by word embedding in Natural Language Processing (NLP) tasks, we treat four different time granularities, month, day of month, hour and minute, as categorical data and map each of them to the corresponding embedding space after transforming them into their one-hot representations. The time embeddings can be formulated as follows:

(2) $$TE\left(T\right)=\sum_{k}^{k\in \left\{M,d,h,m\right\}}W_{k}{one\_hot(T}_{k})$$

where T denotes the timestamps of the input sequence, and $k\in \left\{M,d,h,m\right\}$ indexes the four time granularities of month, day of month, hour, and minute, respectively. $T_{k}$ denotes the categorical time information corresponding to granularity k, one_hot(·) represents the one-hot encoding operation, and $W_{k}$ denotes the learnable embedding matrix for the corresponding temporal granularity.

### 3.3. Network Architecture

We propose a sequence model based on the encoder–decoder architecture for long-term periodic time series prediction. The goal of the encoder is to capture the temporal dependency among input series and form the latent representation of input embeddings mentioned in Section 3.2. This latent representation is then fed to the decoder to model the relation between input and target series. Our decoder design originated from Informer [17], which takes position encodings and time embeddings of the target series and generates predictions conditionalized by the output of the encoder in one iteration. This generative-style decoder has been shown to be more efficient than the traditional autoregressive decoder by Zhou et al.

The encoder and decoder are composed of a series of neural network layers, which can use either GRU [11] or Transformer [16] blocks as the basic computation logic. When using GRU as the inner computation block, each GRU layer in the encoder is initialized to **0** and fed with input embeddings to process the temporal dependency of the input series. The output of the *i*-th GRU layer in the encoder, denoted as $h_{en}^{i}$, is then used to initialize the *i*-th GRU layer in the decoder so that the decoder is conditionalized by the input series. Then, the position encodings and time embeddings of the target series are fed to the decoder to generate final model predictions ${\hat{X}}_{nonlinear}$. Figure 2b gives a clear look at a three-layered model design when using GRU as a building block.

**Figure 3:**
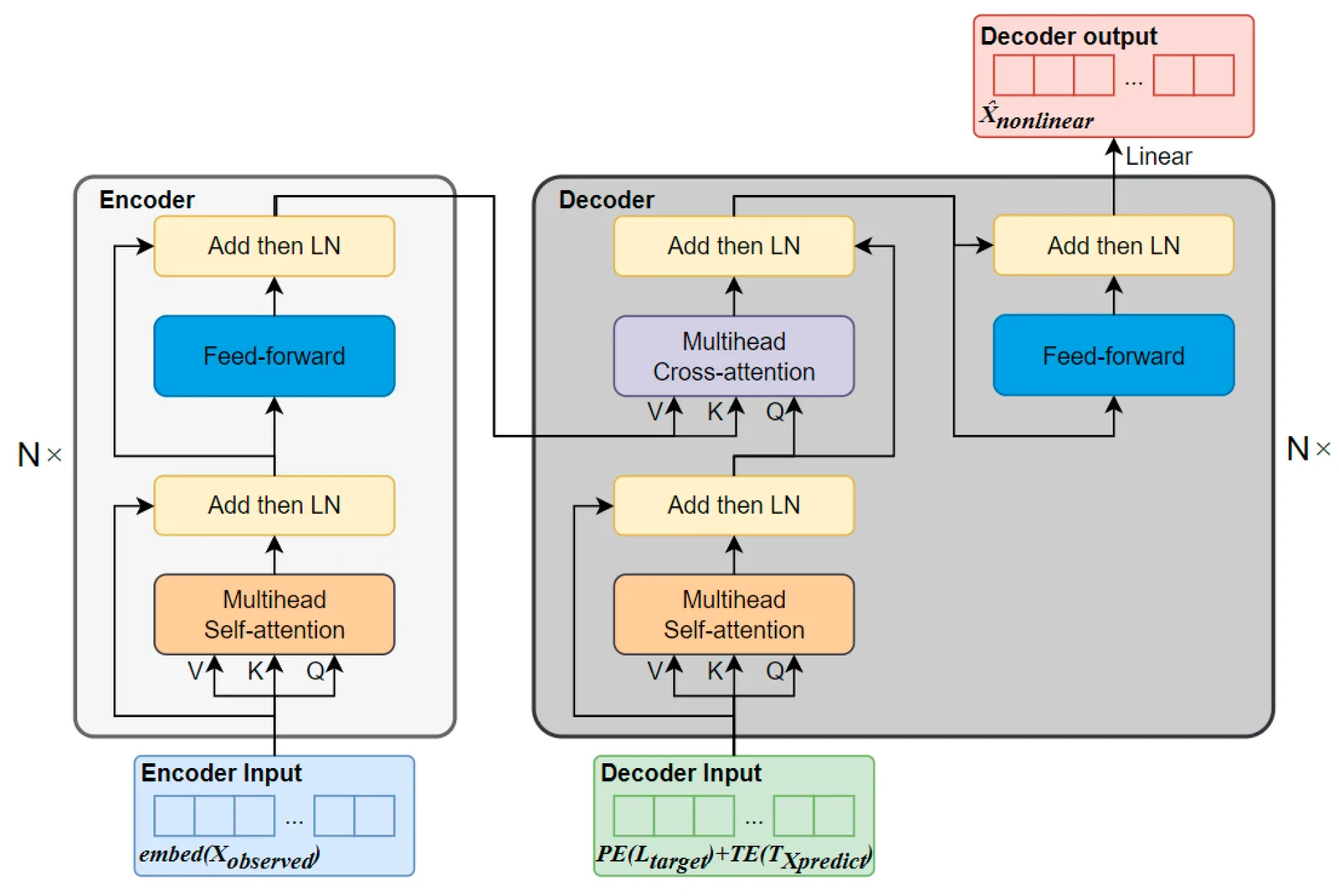
Transformer-based implementation of the proposed encoder–decoder architecture. Q, K, and V denote the query, key, and value representations, respectively; LN denotes layer normalization; and N represents the number of stacked Transformer layers.

We also adopt the Transformer architecture in our realization. When using a Transformer block as a basic building block, the overall design is identical to the vanilla Transformer, except that the decoder only depends on position encodings of $X_{predict}$, time embeddings of $X_{predict}$ and the output of the encoder. The full-length predictions ${\hat{X}}_{nonlinear}$ is generated in a single step, unlike an autoregressive decoder that generates predictions one by one depending on previous predictions. We give a drawing of our model implemented by an N-layer Transformer in Figure 3. In the figure, Q, K, and V denote the query, key, and value representations used in the self-attention and cross-attention modules, respectively, while LN denotes layer normalization. N represents the number of stacked Transformer layers. The encoder input is given by $Embed\left(X_{observed}\right)$, whereas the decoder input consists of the positional encoding $PE\left(L_{target}\right)$ and temporal embedding $TE\left(T_{X_{predict}}\right)$ of the target sequence. The decoder produces the nonlinear prediction ${\hat{X}}_{nonlinear}$ through a final linear projection layer.

### 3.4. Temporal Linear Regression

The deep neural encoder–decoder network in Section 3.3 generates predictions by learning short-term and long-term temporal dependencies between input and output series. We refer to these predictions as the nonlinear component. When observing the error of this nonlinear prediction, it is evident that although the nonlinear characteristics of deep neural networks help capture the seasonality and periodicity of time series, it is not sensitive enough to short-term local variations. As a result, it performs sub-optimally on time series with drastic variations within a short period of time, falling short of the expected accuracy. This phenomenon was pointed out in [14], attributing it to the inherent nonlinear nature of deep learning networks, causing the output range of deep learning networks to be less responsive to the input scale, thus affecting prediction accuracy.

To address these issues, we adopted the approach of LSTNet [14] and designed a bypass linear unit parallel to the neural network. This unit is similar to autoregressive models in traditional time series forecasting methods. However, to maintain the parallelization property of the model, we restrict the prediction of each time step to be a linear combination of past observations of the input series, and perform a linear regression based on these past observations. Note that we only focus on the temporal relation between past and future; the covariances between variables are not considered, so each variable has its own weight matrix. Therefore, we denote this linear unit as temporal linear regression (TLR).

TLR unit models univariate temporal mapping per feature without considering cross-variable covariances. Compared to a full multi-variable linear projection, whose parameters grow quadratically with respect to feature dimension, our independent weight formulation restricts parameter growth strictly to linear complexity. For ultra-large-scale dimension conditions, the proposed method might encounter potential bottlenecks. When the variable dimension scales up significantly, storing high-dimensional distinct linear weight matrices could increase memory utilization during backpropagation. For such cases, channel-independent weight sharing can be adopted to compress the TLR parameter count.

## 4. Experimental Results

### 4.1. Datasets

We used two real-world time series datasets to evaluate the effectiveness of the proposed method in practical applications.

- EC (Electricity Consumption) [29]: This dataset consists of hourly electricity consumption of 321 households, spanning three years from 2012 to 2014. These data points are derived from Smart Meters (Advanced Metering Infrastructure—AMI) installed in households. These meters act as endpoints that measure voltage, current, and phase angle at high frequencies to calculate kilowatt-hour (kWh) usage. It can be used to test the model’s capability in addressing big data challenges inherent in smart grid management, where high-resolution temporal forecasting is critical for demand response, load balancing, and energy efficiency.
- SIC (Speed Index California) [30]: This dataset consists of the average speed index of highways across 10 districts in California. These data points are obtained from the California Department of Transportation, generated by Inductive Loop Detectors (ILDs) embedded in highway lanes, or alternatively, Probe Vehicle Data collected via connected vehicle telemetry. The speed index is used to measure overall traffic congestion across district highways, spanning the eight years from 2015 to 2022. This dataset evaluates the model’s ability to capture both short-term trends, such as sudden congestion or accidents, and long-term trends, such as periodic rush hour patterns. These capabilities are vital for real-time traffic management and autonomous navigation within Intelligent Transportation System (ITS) applications.

While both datasets exhibit clear 24-h daily periodicity, they inherently contain significant real-world noise and non-stationary variations. For example, the SIC dataset contains sudden, highly dynamic speed drops caused by traffic incidents or severe weather. The EC dataset includes fine-grained single-household smart meter telemetry. Both datasets demonstrate some noisy, non-stationary local behaviors in spite of their macroscopic periodicity.

As we focus on periodic time series forecasting, in order to examine the existence of cyclic repeating patterns in time series data, we randomly select 10 variables from the variables in the EC dataset for autocorrelation analysis, while all 9 variables in the SIC dataset are used due to its relatively small dimensionality. This analysis is intended to provide a visual characterization of the periodic properties of the datasets. The autocorrelation function ${ACF}_{X}\left(\tau \right)$ of series X can be formulated as follows:

(3) $${ACF}_{X}\left(\tau \right)=\frac{E\left[\left(X_{t}-\mu_{t}\right)\left(X_{t-\tau }-\mu_{t-\tau }\right)\right]}{\sigma_{t}\sigma_{t-\tau }}$$

where $X_{t}$ and $X_{t-\tau }$ denote the observations of the time series X at time steps t and t−τ, respectively; τ represents the time lag; $\mu_{t}$ and $\mu_{t-\tau }$ denote the corresponding mean values; $\sigma_{t}$ and $\sigma_{t-\tau }$ denote the corresponding standard deviations; and $E\left[\cdot \right]$ denotes the expectation operator.

**Figure 4:**
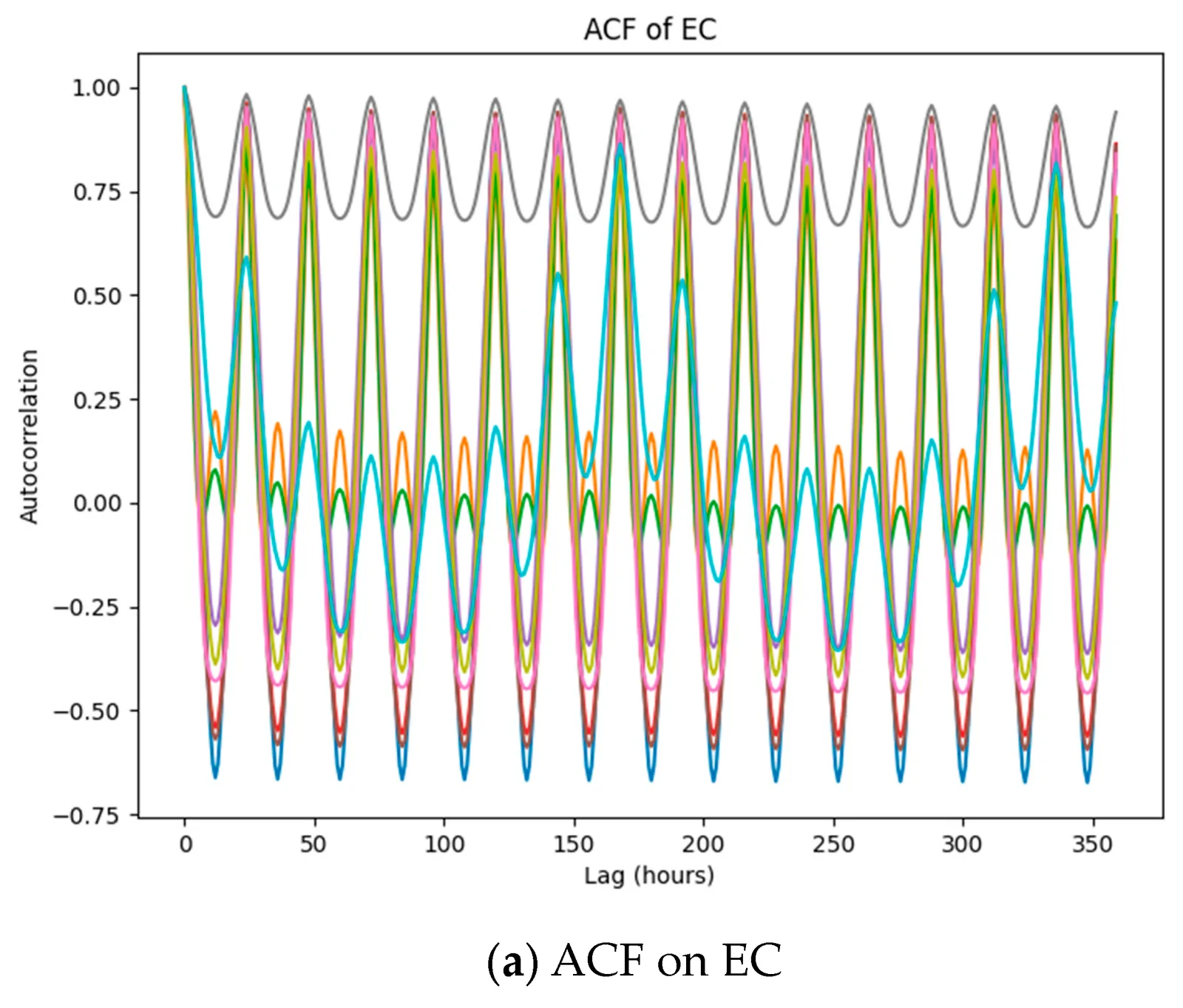

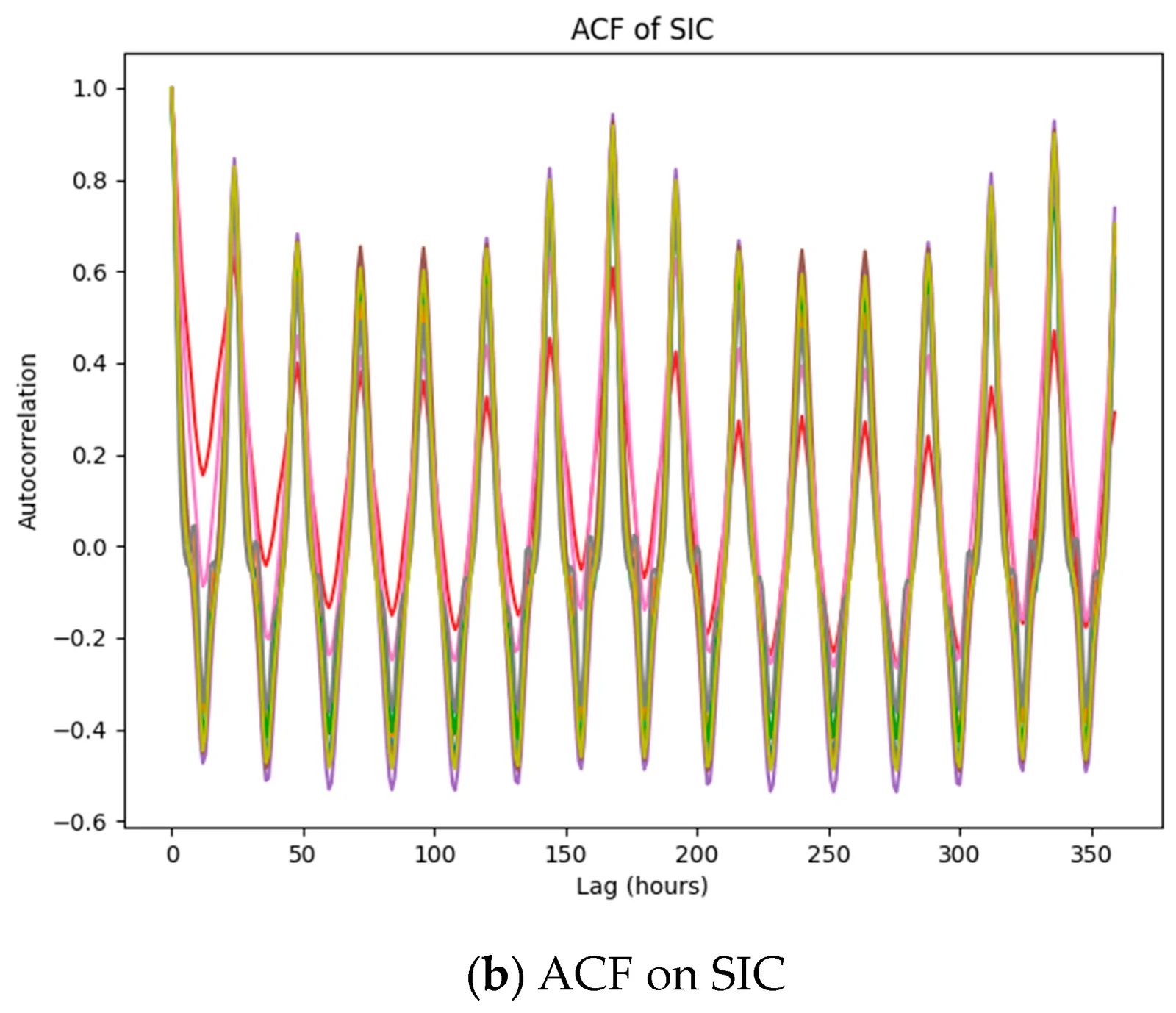
Autocorrelation on each dataset.

**Figure 5:**
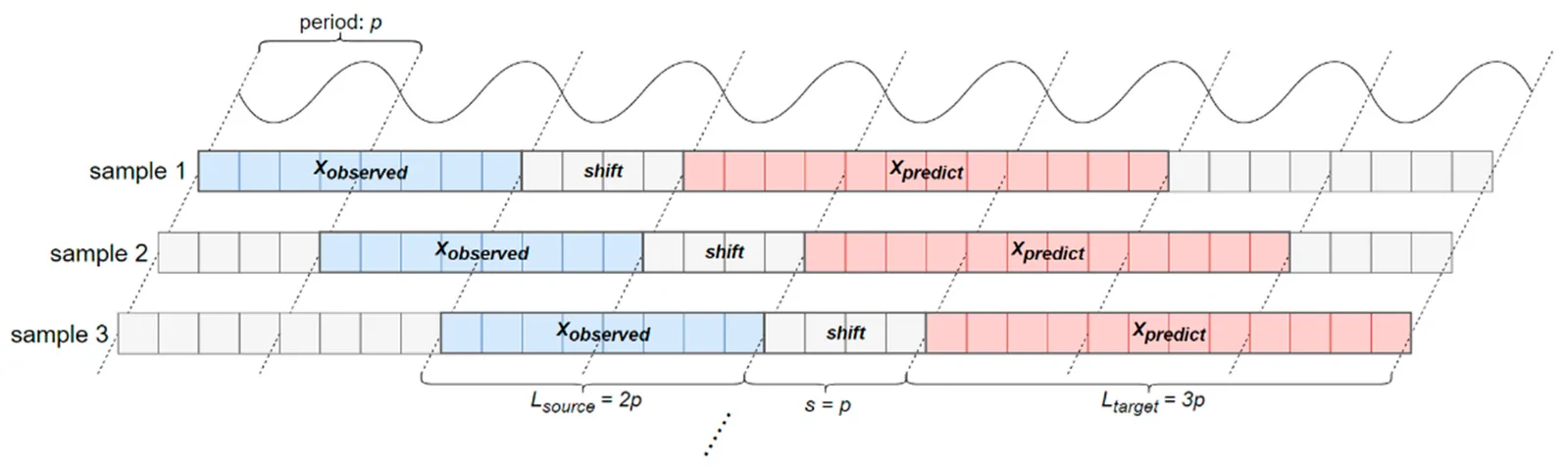
Preparation of feeding sequences.

After examining Figure 4a,b, we found that two datasets exhibit obvious 24-h short-term periodicity. To preserve these repeating structures, we slice the time series with a stride of 24 h when preparing feeding sequences for the training process, that is, predicting the target sequence on a daily basis. We visualize the overall sequence preparation process in Figure 5.

Lastly, we split each dataset into training, validation and test sets by an 8:1:1 ratio in chronological order and standardize the data distribution before training. Three metrics, Mean Squared Error (MSE), mean absolute error (MAE) and Pearson’s correlation coefficient (Corr), are considered in the experiments. Note that lower MSE and MAE values are better, while a higher Corr value is better. For baseline comparisons, we report MSE (magnitude accuracy) and Corr (temporal phase alignment) to highlight the model’s structural advantage in tracking sequence dynamics without overcrowding the tables. In the ablation study, we report MSE, MAE, and Corr across all prediction horizons. While MSE quadratically penalizes larger deviations, MAE provides a linear and robust evaluation of pointwise absolute scale errors. Reporting both MSE and MAE in the ablation study allows us to effectively monitor error propagation behaviors without over-penalizing or masking intermittent fluctuations over long horizons. When performing comparisons with state-of-the-art methods, we present only MSE and MAE statistics since the recent SOTA literature rarely reports Corr values.

### 4.2. Baselines

We have selected four baseline models for quantitative comparison. Selected methods involve a naïve method, heuristic method, classic statistical model and deep learning model. All four baseline models are listed below.

- **Persistence** uses the last 24 h of observations as the prediction for every day in the future.
- **MA** is the abbreviation for Periodic Moving Average, calculating the average historical values for each hour over multiple days, forming a 24-h prediction. This prediction is then copied multiple times as the prediction for multiple days.
- **AR** stands for autoregressive model, assuming that the current value has a linear relationship with historical observations plus a constant term and random error. During the prediction phase, the previous time step prediction is used as input to generate the target sequence step by step. As AR is a univariate prediction model, in the case of multivariate applications, we build several independent AR models to predict each variable.
- **LSTNet** [14] is a deep learning network architecture that combines different network units to learn the long-term and short-term features of input series.

### 4.3. Setup

All models are trained and tested on a single RTX 2080 Ti 12 GB GPU (NVIDIA Corporation, Santa Clara, CA, USA) and implemented with TensorFlow. All learning models use Mean Squared Error (MSE) as the loss function and use Adam as the optimizer. The learning rate is set to 0.001, with β1 = 0.9 and β2 = 0.999, following the standard Adam configuration. The batch size is fixed at 32 to maintain a consistent training setting across all learning models, and early stopping is employed to avoid overfitting. For architecture-related hyperparameters, we perform grid search on the validation set of each dataset.

### 4.4. Results

Table 1 and Table 2 summarize experiment results of output length and shift at different settings on both datasets. In general, the longer the output length or shift is, the harder the forecasting task is. As our proposed model can use GRU or self-attention as the basic operation, we denote the model using GRU as “Ours-GRU”, and the model using self-attention as “Ours-SA”.

**Output length:** Under this setting, we fix the input length $L_{source}$ to 168, and fix shift to 0. We prolong the output length $L_{target}$ to be $\left\{24,\;96,\;168,\;240,\;312\right\}$ to test how each model performs as $L_{target}$ increase. On the EC dataset, the best-performing model overall is Ours-GRU, with an average MSE of 0.1582 for all output lengths, leading by about 22% compared to the best baseline method, LSTNet, with 0.2034. Ours-SA also has an average MSE of 0.1694, still leading LSTNet by about 16%. Similar results are also shown on the SIC dataset, where Ours-GRU has an average MSE of 0.1162 for all output lengths, leading the best LSTNet with 0.1394 by about 16%. Ours-SA has an average MSE of 0.1278, which is better than LSTNet by about 8%.

**Shift:** Under this setting, we fix the input length $L_{source}$ to 168, and fix the output length $L_{target}$ to 168. We prolong shift s to be $\left\{24,\;96,\;168,\;240,\;312\right\}$ to test how every model performs as the temporal distance between the input sequence and the target sequence increases. Note that because of the discontinuity between input and output series, AR was left out of this section. Similar to the results shown in output length experiments, on the EC dataset, both variations of our proposed method outperformed the other three baseline methods. The best-performing baseline method, LSTNet, had an average MSE of 0.2638. Ours-GRU achieved 0.2172, surpassing LSTNet by approximately 17%, and Ours-SA achieved 0.2222, winning over LSTNet by approximately 15%. On the SIC dataset, Ours-GRU had an average MSE of 0.1274, which was about 20% better than LSTNet, which had an MSE of 0.161.

From the experiments, we can observe that Ours-GRU generally outperforms Ours-SA. While self-attention (SA) excels at global receptive fields, it suffers from attention dispersion when applied to continuous time series, treating time points with pairwise soft weights that are vulnerable to dynamic noise. Ours-GRU leverages the continuous state transition of recurrent units. This acts as a robust inductive bias that preserves local temporal momentum and smooths high-frequency fluctuations. For step-by-step state propagation in time-series encoding, GRU maintains tighter trajectory cohesion than the noisy, unconstrained attention weights in Ours-SA.

To further assess the computational efficiency of the proposed model, we measured the wall-clock training and inference times of Ours-GRU on the EC and SIC datasets using the same computational environment described above. For the setting with $L_{target}$ = 168, Ours-GRU required 130.12 s for 56 training epochs on EC and 188.86 s for 78 epochs on SIC. The corresponding inference times were 3.27 s for 14 test samples and 1.12 s for 40 test samples, respectively. For the setting with shift = 168, the training times were 135.89 s for 54 epochs on EC and 118.36 s for 49 epochs on SIC, while the corresponding inference times were 3.27 s for 13 test samples and 1.14 s for 39 test samples, respectively. The number of training epochs differs across experiments because early stopping is employed during training, causing the optimization process to terminate at different epochs according to the validation loss.

### 4.5. Ablation Study

We conduct ablation experiments on Ours-GRU by removing components one at a time in Ours-GRU. First, we name these variants as follows.

- **Ours-GRU w/o TE**: The Ours-GRU model without time embeddings.
- **Ours-GRU w/o LR**: The Ours-GRU model without temporal linear regression.
- **Ours-GRU w/o LR & TE**: The Ours-GRU model without both time embeddings and temporal linear regression.

Table 3 gives a summary of each component’s contributions on each dataset. On the EC dataset, using time embeddings helps Our-GRU achieve an average MSE improvement of nearly 6% compared to Ours-GRU w/o TE. Using temporal linear regression, on the other hand, helps Our-GRU improve average MSE by nearly 55% compared to Ours-GRU w/o LR. On the SIC dataset, the improvement brought by time embeddings is even more significant, with Ours-GRU having an average MSE improvement of nearly 13% compared to Ours-GRU w/o TE. At the same time, it also alleviates the decrease in accuracy caused by increasing output length. The improvement brought by temporal linear regression is a 16% average MSE reduction compared to Ours-GRU w/o LR. To verify implementation stability, these experiments were conducted across five independent random seeds. The results demonstrate extremely small standard deviations, confirming the high stability and robustness of our approach.

Time series in EC and SIC datasets exhibit strong multi-periodic seasonality. Standard recurrent backbones process inputs strictly sequentially, which can lead to phase drift when predicting extended future horizons. TE explicitly maps raw timestamps into high-dimensional cyclic representations. This provides the GRU and Informer decoder with absolute phase anchors. It injects absolute temporal coordinates into the hidden states, enabling the model to disambiguate identical numerical load levels that occur during different operational phases. Without TE, purely autoregressive or recurrent backbones struggle to differentiate between identical magnitude values occurring at different operational states. Therefore, TE acts as a global periodic phase shift that aligns daily morning/evening consumption spikes across 321 electricity series. Consequently, removing TE (Ours-GRU w/o TE) leads to phase misalignment and increased residual variance during peak transition hours. Also note that we can observe that TE yields a greater impact on SIC than on the EC dataset from the ablation study. This disparity stems from the inherent domain properties of the two benchmarks. Electricity (EC) exhibits relatively smoother, self-sustaining daily continuity where recurrent states can partially infer temporal progression from raw load shapes. Conversely, the SIC dataset contains sharper, schedule-driven operational transitions governed by clock time. TE provides absolute phase coordinates (Hour-of-Day, Day-of-Week). For SIC, raw historical trends alone are insufficient to signal calendar shifts. Without TE, the model suffers severe phase misalignment during long-horizon forecasts on highly schedule-dependent datasets like SIC.

The LR branch operates as an explicit baseline trend projector. It contributes most significantly during abrupt baseline transitions (e.g., day–night step changes) and steady-state off-peak periods, where pure neural activations can introduce high-frequency drift. Temporally, LR provides maximum stability during steady off-peak intervals and overall baseline transitions, preventing the recurrent state from accumulating nonlinear prediction drift. Also, LR captures the dominant trend, unburdening the GRU module so it can allocate its parameter capacity toward modeling stochastic, nonlinear surges. As a result, among the 321 electricity variables, LR contributes most notably to high-magnitude, low-variance industrial channels whose load behavior is dominated by relatively consistent routines. Conversely, highly unstable residential channels rely more heavily on the nonlinear GRU path.

**Figure 6:**
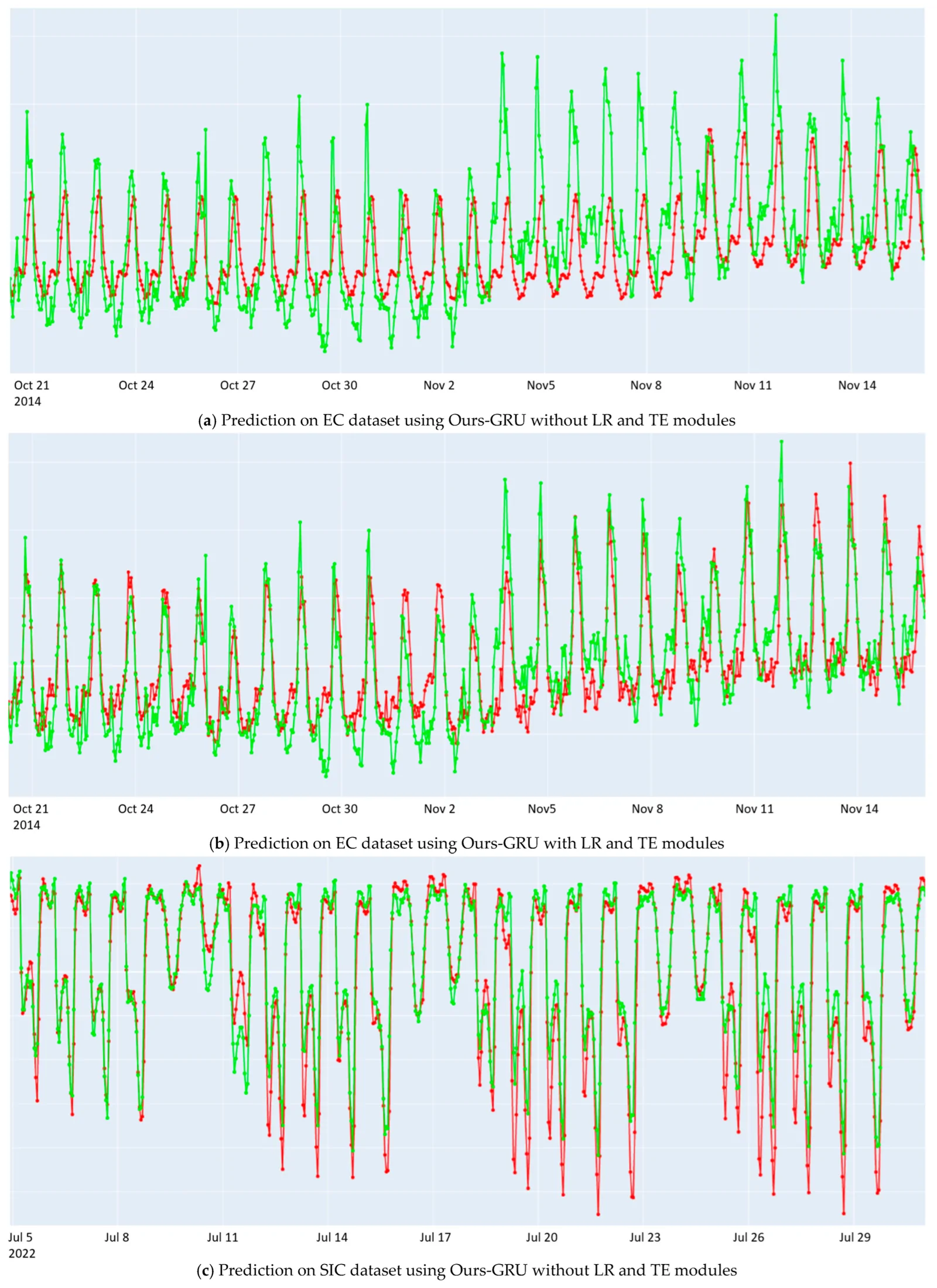

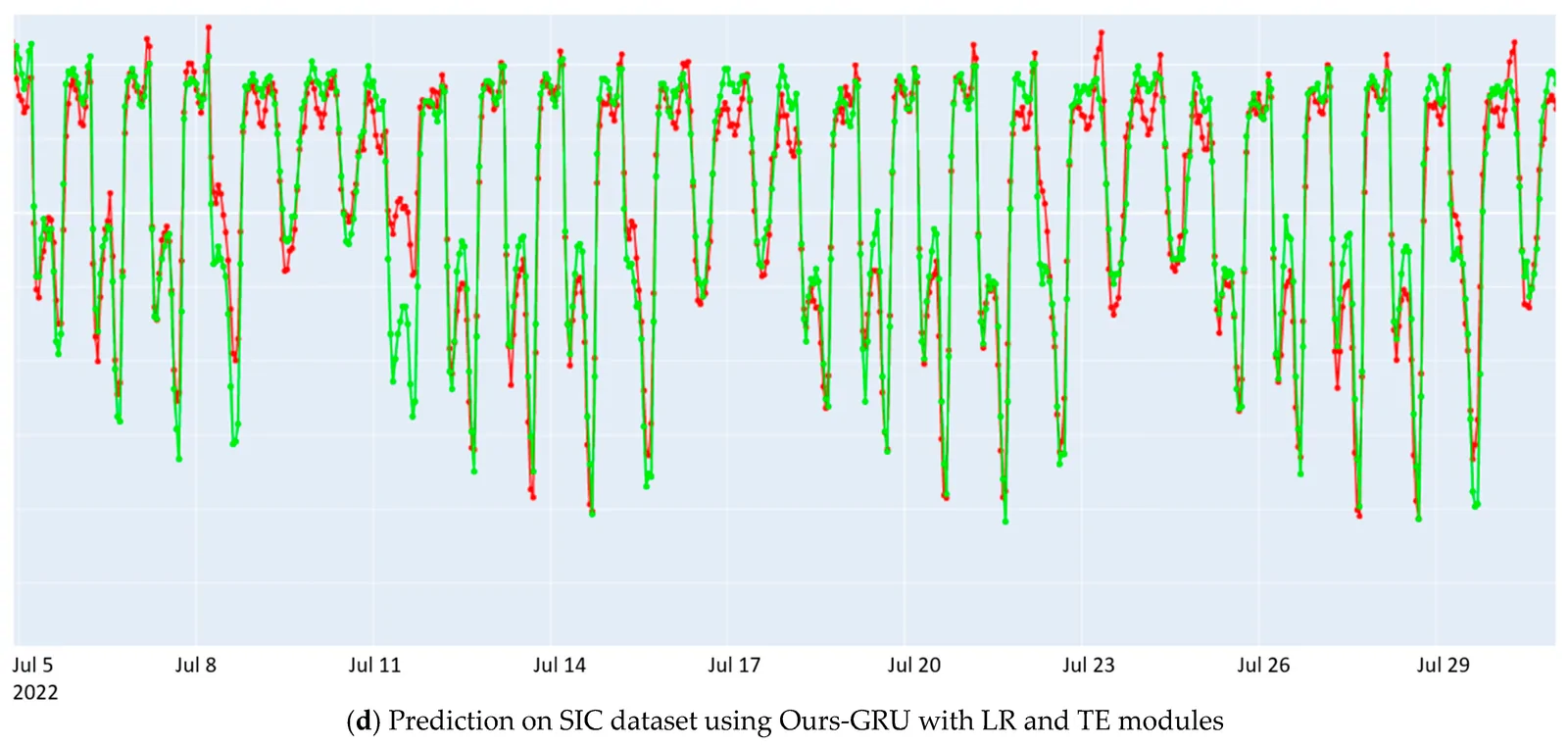
Visualization of time series prediction using Ours-GRU on different datasets.

Figure 6 visualizes the prediction results on the EC and SIC datasets using line plots to provide a more intuitive presentation. All experiments were conducted under a standardized setting with an input sequence length of 168, an offset of 0, an output sequence length of 168, and a GRU-based backbone model. We observe the qualitative impact of the TE and the LR on forecasting performance across both datasets. In Figure 6, the green dots and lines represent the ground truth, while the red dots and lines denote the predicted values. Specifically, Figure 6a illustrates the prediction results on the EC dataset without the TE and LR modules, whereas Figure 6b displays the results obtained on the EC dataset after incorporating both TE and LR. Comparing Figure 6a,b, it is evident that without TE and LR, the predictions in Figure 6a exhibit noticeable underestimation, demonstrating less sensitivity to both peaks and valleys of the original sequence compared to the full model. Conversely, the forecasting results in Figure 6b show significant improvements in fitting local extrema across the sequence. Figure 6c depicts the prediction results on the SIC dataset without the TE and LR modules, while Figure 6d presents the results on the SIC dataset with TE and LR integrated. As shown in Figure 6c, the model without TE and LR substantially overestimates the downward spikes compared to the ground truth. Incorporating TE and LR effectively mitigates this issue. As demonstrated in Figure 6d, the full model achieves obvious improvements in capturing local extrema relative to Figure 6c.

### 4.6. Comparison with State-of-the-Art Methods

To demonstrate the effectiveness of the proposed framework, Table 4 presents a comparative evaluation of the proposed method against state-of-the-art models. Note that since many state-of-the-art methods do not report experimental results on the SIC dataset, we select the widely adopted Electricity (EC) dataset as the main benchmark for comparison. Also, not all studies provide complete benchmark evaluations across all prediction horizons. Therefore, we anchor our main comparative analysis on the Electricity (EC) benchmark with a standard prediction horizon of $L_{target}=96$. Note that in Table 4, the statistics of Autoformer, Pyraformer, FEDformer, NLinear, Scaleformer, and PatchTST are cited from [31]. The statistics of Informer and TimesNet are cited from [23]. The statistics of Crossformer and FCP-Former are cited from [28]. The statistics of MSFformer are cited directly from its original paper [27]. We have noticed that MSFformer reported a significantly higher MSE of 0.4698 on the EC dataset. We presume that the reason for this unreasonably high error primarily stems from the absence of proper instance normalization (e.g., Reversible Instance Normalization, RevIN) used in other SOTA methods in its original experimental setup.

Overall, Ours-GRU achieves superior predictive accuracy in terms of Mean Squared Error (MSE). Specifically, compared to classic Transformer variants such as Informer and Pyraformer, our method exhibits a substantial error reduction exceeding 40%~60%. While early Transformers suffer from severe information dilution and quadratic attention noise across high-dimensional channel inputs, Ours-GRU leverages recurrent gating mechanisms to effectively preserve long-term autoregressive continuity and suppress localized variance. Compared to recent strong models including TimesNet, PatchTST, and FCP-Former, Ours-GRU delivers the lowest MSE. NLinear relies on a fixed linear projection matrix, which inherently fails to model the complex, nonlinear load surges characteristic of power grid data. This limitation generates large residual peaks that are heavily penalized under MSE. In contrast, Ours-GRU incorporates nonlinear gating mechanisms and hidden state updates, allowing for dynamic temporal dependency modeling that improves both localized spike fitting and overall absolute deviation. TimesNet relies on Fast Fourier Transform (FFT) to transform 1D time series into 2D spaces based on dominant periodicities. However, electricity consumption data contains frequent non-periodic load surges. While FFT-based frequency selection smoothing struggles to capture abrupt nonlinear spikes, the step-by-step hidden state updates in Ours-GRU retain continuous temporal responsiveness. Scaleformer employs an iterative multi-scale refining pipeline that progressively enhances coarse predictions. This hierarchical setup is susceptible to error propagation, where initial trajectory misalignments at coarse scales are cascaded and magnified down to fine-grained forecasting steps. In contrast, Ours-GRU operates within a unified, end-to-end temporal recurrent framework, eliminating multi-stage error accumulation and yielding tighter absolute trajectory alignment. Crossformer utilizes a two-stage attention mechanism to explicitly capture both cross-time and cross-dimension dependencies. When scaled to the EC dataset, cross-channel attention introduces matrix noise and parameter overfitting due to sparse channel correlations. Ours-GRU bypasses high-dimensional cross-channel attention noise, focusing its capacity on capturing robust, localized temporal dynamics without suffering from over-parameterization degradation. While patch-based methods reduce complexity by grouping temporal steps into sub-series tokens, hard patch segmentation sometimes introduces boundary discontinuities between adjacent tokens and loses fine-grained step-level details. Ours-GRU maintains continuous recurrent state transitions, effectively eliminating artificial boundary steps. This continuous trajectory preservation reduces prediction outliers, leading to a lower MSE. While FCP-Former achieves a lower MAE, Ours-GRU demonstrates a highly competitive performance balance. Mathematically, MSE is more sensitive to larger prediction inconsistencies due to its quadratic penalty, whereas MAE reflects average absolute linear deviations. FCP-Former’s frequency-domain compensation appears particularly effective at smoothing overall baseline trajectories, contributing to its lower MAE. Conversely, the recurrent state transitions in Ours-GRU provide slight advantages in modeling localized nonlinear surges, which helps mitigate extreme residual peaks and leads to improved overall variance control.

## 5. Conclusions

In this paper, we propose a versatile hybrid deep learning framework designed for long-term forecasting of real-world periodic time series. By preserving recurring temporal structures during sequence sampling and decoupling trend–residual dynamics, our framework mitigates the complexity of long-range dependencies. Extensive empirical evaluations and ablation studies validate the distinct functional boundaries and quantitative benefits of each core innovation. The proposed model substantially outperforms baseline architectures across multi-step scenarios, achieving top-tier accuracy on real-world benchmarks. The proposed model also outperforms state-of-the-art methods on the EC dataset at $L_{target}=96$ in terms of Mean Squared Error. Specifically, the temporal linear regression (LR) bypass module effectively captures global baseline transitions and increases local prediction response. Integrating global datetime information via Temporal Embeddings (TEs) provides crucial phase anchoring. This eliminates phase drift across extended forecasting horizons (up to $L_{target}=312$), significantly improving local peak and valley fitting without trajectory overshoot or underestimation. For future research, we identify several potential directions to extend our work. A primary direction is to integrate special event encodings, such as holidays and festivals, into the proposed time embeddings. Second, we aim to explore alternative attention-based sequence backbones to further test modular switching. Finally, the temporal linear regression module, which currently exhibits a linear scaling of parameters relative to the number of variables, can be further refined to improve efficiency.

## Figures and Tables

**Table 1:** Experimental results for different output lengths on two datasets. Bold values indicate the best performance for each method.

Models		Persistence		MA		AR		LSTNet		Ours-GRU		Ours-SA	
Datasets	$L_{target}$	MSE	Corr	MSE	Corr	MSE	Corr	MSE	Corr	MSE	Corr	MSE	Corr
EC	24	0.259	0.838	0.183	0.867	0.215	0.861	0.138	0.900	**0.133**	**0.903**	0.147	0.895
	96	0.344	0.768	0.195	0.858	0.293	0.805	0.184	0.868	**0.154**	**0.888**	0.172	0.875
	168	0.312	0.802	0.206	0.849	0.364	0.792	0.184	0.870	**0.165**	**0.879**	0.168	0.877
	240	0.325	0.793	0.21	0.845	0.866	0.775	0.235	0.852	**0.166**	**0.875**	0.177	0.866
	312	0.335	0.786	0.215	0.842	0.946	0.681	0.276	0.835	**0.173**	**0.871**	0.183	0.865
SIC	24	0.383	0.803	0.318	0.816	0.282	0.844	0.103	0.948	**0.096**	**0.951**	0.098	0.949
	96	0.687	0.640	0.334	0.806	0.428	0.746	0.131	0.935	**0.115**	**0.941**	0.121	0.939
	168	0.597	0.689	0.344	0.801	0.427	0.749	0.148	0.928	**0.119**	**0.938**	0.131	0.934
	240	0.628	0.673	0.35	0.797	0.452	0.734	0.151	0.928	**0.127**	**0.936**	0.140	0.929
	312	0.648	0.663	0.353	0.795	0.471	0.723	0.165	0.926	**0.124**	**0.936**	0.149	0.924

**Table 2:** Experimental results of the shift on all datasets. Bold values indicate the best performance for each method.

Models		Persistence		MA		LSTNet		Ours-GRU		Ours-SA	
Datasets	s	MSE	Corr	MSE	Corr	MSE	Corr	MSE	Corr	MSE	Corr
EC	24	0.322	0.796	0.214	0.844	0.200	0.863	**0.168**	**0.875**	0.190	0.862
	96	0.344	0.782	0.234	0.832	0.280	0.812	0.215	0.842	**0.211**	**0.845**
	168	0.366	0.769	0.251	0.823	0.268	0.817	**0.210**	**0.847**	0.228	0.834
	240	0.381	0.761	0.265	0.818	0.248	**0.836**	0.243	0.827	**0.237**	0.833
	312	0.396	0.756	0.280	0.814	0.323	0.799	0.250	**0.832**	**0.245**	0.828
SIC	24	0.608	0.684	0.351	0.797	0.142	0.929	**0.113**	**0.942**	0.142	0.929
	96	0.629	0.674	0.365	0.788	0.152	0.923	**0.125**	**0.936**	0.157	0.919
	168	0.647	0.666	0.374	0.783	0.177	0.918	**0.131**	**0.932**	0.167	0.915
	240	0.649	0.664	0.380	0.779	0.163	0.920	**0.135**	**0.934**	0.163	0.917
	312	0.655	0.661	0.383	0.778	0.171	0.913	**0.133**	**0.934**	0.175	0.912

**Table 3:** Results of ablation experiments on all datasets. Bold values indicate the best performance for each setting.

Models		Ours-GRU			Ours-GRU w/o TE		
Dataset	$L_{target}$	MSE	MAE	Corr	MSE	MAE	Corr
EC	24	**0.133 ± 0.0006**	**0.256 ± 0.0006**	**0.903 ± 0.0004**	0.147 ± 0.0009	0.270 ± 0.0009	0.894 ± 0.0008
	96	**0.154 ± 0.0006**	**0.263 ± 0.0006**	**0.888 ± 0.0005**	0.168 ± 0.0013	0.283 ± 0.0015	0.877 ± 0.0013
	168	**0.165 ± 0.0008**	**0.280 ± 0.0009**	**0.879 ± 0.0008**	0.170 ± 0.0018	0.289 ± 0.0018	0.875 ± 0.0019
	240	**0.166 ± 0.0010**	**0.282 ± 0.0012**	**0.875 ± 0.0011**	0.175 ± 0.0015	0.294 ± 0.0016	0.869 ± 0.0016
	312	**0.173 ± 0.0017**	**0.290 ± 0.0016**	**0.871 ± 0.0016**	0.179 ± 0.0020	0.298 ± 0.0021	0.867 ± 0.0020
SIC	24	**0.096 ± 0.0008**	**0.147 ± 0.0008**	**0.951 ± 0.0008**	0.098 ± 0.0011	0.150 ± 0.0013	0.949 ± 0.0012
	96	**0.115 ± 0.0010**	**0.161 ± 0.0011**	**0.941 ± 0.0011**	0.132 ± 0.0015	0.171 ± 0.0016	0.931 ± 0.0016
	168	**0.119 ± 0.0012**	**0.164 ± 0.0012**	**0.938 ± 0.0013**	0.145 ± 0.0020	0.179 ± 0.0020	0.926 ± 0.0019
	240	**0.127 ± 0.0014**	**0.170 ± 0.0013**	**0.936 ± 0.0013**	0.147 ± 0.0018	0.181 ± 0.0019	0.925 ± 0.0017
	312	**0.124 ± 0.0012**	**0.168 ± 0.0012**	**0.936 ± 0.0013**	0.150 ± 0.0022	0.183 ± 0.0022	0.923 ± 0.0021
Models		Ours-GRU w/o LR			Ours-GRU w/o LR & TE		
Dataset	$L_{target}$	MSE	MAE	Corr	MSE	MAE	Corr
EC	24	0.292 ± 0.0018	0.329 ± 0.0020	0.837 ± 0.0019	0.310 ± 0.0025	0.391 ± 0.0025	0.829 ± 0.0024
	96	0.355 ± 0.0016	0.428 ± 0.0016	0.800 ± 0.0018	0.366 ± 0.0028	0.439 ± 0.0029	0.789 ± 0.0027
	168	0.372 ± 0.0020	0.466 ± 0.0018	0.790 ± 0.0020	0.376 ± 0.0027	0.470 ± 0.0027	0.778 ± 0.0026
	240	0.382 ± 0.0014	0.485 ± 0.0015	0.760 ± 0.0015	0.392 ± 0.0023	0.493 ± 0.0024	0.752 ± 0.0023
	312	0.377 ± 0.0026	0.471 ± 0.0028	0.773 ± 0.0025	0.385 ± 0.0030	0.488 ± 0.0031	0.770 ± 0.0029
SIC	24	0.111 ± 0.0022	0.157 ± 0.0023	0.948 ± 0.0022	0.119 ± 0.0031	0.162 ± 0.0032	0.942 ± 0.0029
	96	0.137 ± 0.0019	0.176 ± 0.0019	0.933 ± 0.0020	0.143 ± 0.0033	0.178 ± 0.0034	0.932 ± 0.0032
	168	0.144 ± 0.0022	0.180 ± 0.0023	0.928 ± 0.0023	0.156 ± 0.0028	0.199 ± 0.0029	0.926 ± 0.0028
	240	0.151 ± 0.0020	0.185 ± 0.0021	0.933 ± 0.0020	0.160 ± 0.0032	0.235 ± 0.0033	0.921 ± 0.0031
	312	0.154 ± 0.0023	0.189 ± 0.0024	0.929 ± 0.0023	0.179 ± 0.0034	0.254 ± 0.0035	0.913 ± 0.0033

**Table 4:** Comparison with state-of-the-art methods on EC dataset under $L_{target}=96.$ Bold values indicate the best performance, and underlined values indicate the second-best performance.

Model	Publication/Year	MSE	MAE
LSTNet	ACM SIGIR/2018	0.184	0.302
Informer	AAAI/2021	0.274	0.368
Autoformer	NeurIPS/2021	0.196	0.313
FEDformer	ICML/2022	0.186	0.302
Pyraformer	ICLR/2022	0.386	0.449
NLinear	AAAI/2023	0.185	0.266
TimesNet	ICLR/2023	0.168	0.272
Scaleformer	ICLR/2023	0.182	0.297
Crossformer	ICLR/2023	0.219	0.314
PatchTST	ICLR/2023	0.180	0.264
MSFformer	Mathematics/2024	0.469	0.264
FCP-Former	Sensors/2025	0.156	**0.250**
Ours-GRU	-	**0.154**	0.263

## Data Availability

No new data were created or analyzed in this study. Data sharing is not applicable to this article.
